# A longitudinal assessment of periodontal disease in 52 miniature schnauzers

**DOI:** 10.1186/1746-6148-10-166

**Published:** 2014-09-01

**Authors:** Mark D Marshall, Corrin V Wallis, Lisa Milella, Alison Colyer, Andrew D Tweedie, Stephen Harris

**Affiliations:** 1 The WALTHAM Centre for Pet Nutrition, Melton Mowbray, Leicestershire LE14 4RT, UK; 2 The Veterinary Dental Surgery, 53 Parvis Road, Byfleet, Surrey KT14 7AA, UK

**Keywords:** Dog, Gingivitis, Periodontitis, Periodontal disease, Miniature schnauzer

## Abstract

**Background:**

Periodontal disease (PD) is the most widespread oral disease in dogs and has been associated with serious systemic diseases. The disease is more prevalent in small breeds compared to large breeds and incidence increases with advancing age. In prevalence studies 84% of beagles over the age of 3 and 100% of poodles over the age of 4 were diagnosed with PD. Current knowledge of the rate of progression of PD is limited. The objective of this study was to determine the rate of PD progression in miniature schnauzers, an at risk small breed of dog.

Dogs (n = 52, age 1.3-6.9 years) who had received a regular oral care regime prior to this study were assessed for levels of gingivitis and periodontitis around the whole gingival margin in every tooth under general anaesthetic. Assessments were conducted approximately every six weeks for up to 60 weeks following the cessation of the oral care regime.

**Results:**

All of the 2155 teeth assessed entered the study with some level of gingivitis. 23 teeth entered the study with periodontitis, observed across 12 dogs aged between 1.3 and 6.9 years. 35 dogs had at least 12 teeth progress to periodontitis within 60 weeks. Of the teeth that progressed to periodontitis, 54% were incisors. The lingual aspect of the incisors was significantly more likely to be affected (*p* < 0.001). The severity of gingivitis in periodontitis-affected teeth was variable with 24% of the aspects affected having very mild gingivitis, 36% mild gingivitis and 40% moderate gingivitis. Periodontitis progression rate was significantly faster in older dogs. Only one dog (age 3.5) did not have any teeth progress to periodontitis after 60 weeks.

**Conclusions:**

This is the first study to have assessed the progression rate of periodontitis in miniature schnauzers and highlights that with no oral care regime, the early stages of periodontitis develop rapidly in this breed. An oral care regime and twice yearly veterinary dental health checks should be provided from an early age for this breed and other breeds with similar periodontitis incidence rates.

## Background

Periodontal disease (PD) is the most widespread oral disease in dogs and prevalence estimates of 44%, 56%, 60% and 63.6% have been reported [1-4].

The incidence and severity of the disease has been shown to increase with age [2,4,5]. In a study of poodles, 90% under 4 years of age and all dogs older than four years were reported to have at least one tooth with periodontitis [5]. Kortegaard *et al.* observed that all research beagle dogs in their study, regardless of age, had gingivitis diagnosed by bleeding on probing [2]. In that study 20% of dogs aged between 1 and 2 years had clinical attachment loss, increasing to 61% of dogs aged between 2 and 3 years and 84% of dogs aged 3 years or over [2]. In an earlier study of 162 dogs of various breeds, 37% of dogs aged younger than 2 years were affected with marginal periodontitis, rising to 55.2% of dogs aged 3–5 years and 82.3% of dogs aged six years or older [4].

Periodontitis has also been reported to be more prevalent in small breeds compared to large breeds [3,4,6]. In addition, brachycephalic breeds and dogs with tooth overcrowding have been reported to be especially vulnerable to developing the advanced stages of the disease [7]. The number of affected teeth has also been shown to vary considerably between dogs of the same breed [2,5].

Disparities in reported prevalence estimates are likely due to differences in age and breed compositions of the study groups. The criteria used to establish the diagnosis of PD will also affect the reported prevalence estimates. Some studies report gingivitis, plaque and calculus as disease conditions [3,8] whilst others include clinical attachment loss and periodontal probing depth as measures of periodontitis [2,5]. Bone loss during post-mortem examination [4] or determined by dental radiology [1] have also been utilized to determine diagnosis. In addition, the thresholds used for diagnosing pathological pockets and clinical attachment loss differ between studies. For example, Kyllar & Witter [3] used probing depths of between 1 and 2.5 mm to determine early periodontitis in various breeds of dog, whereas Hoffmann & Gaengler [5] classified a periodontal pocket of 3-5 mm as slight periodontitis in poodles. The sample sizes studied also vary which will affect the precision of the estimates obtained and some of the studies have only examined a subset of the dentition [3,9]. These variations are not unique to veterinary dentistry. There are also many indices used to assess the clinical status of human patients in periodontology research studies [10,11]. The aforementioned differences make it difficult to compare studies and may risk overstating or understating the problem.

The aim of this study was to determine the incidence and progression of gingivitis and periodontitis in miniature schnauzer dogs based on full-mouth examinations using periodontal probing depth, gingival recession and furcation exposure as indicators of clinical attachment loss.

## Results

### Number of assessments

The number of dogs and number of teeth assessed at each measurement are reported in Table 1. Dogs were assessed at 6 week intervals (+/− 1 week) for between 3 and 11 times over the course of the 60 week study. One dog was not assessed at the first 6 week time point due to health reasons unrelated to the study. The number of teeth within a dog that progressed to periodontitis ranged from 0 to 21. Some dogs had less than 12 teeth affected at one assessment period meaning that they stayed on trial for one more 6 week period but by the time of the next assessment up to 21 teeth were affected.

**Table 1:** Number of animals and number of teeth assessed at each time point

**Time from trial start (weeks +/− 1 week)**	**0**	**6**	**12**	**18**	**24**	**30**	**36**	**42**	**48**	**54**	**60**
**Total animals assessed**	52	51	52	51	49	43	38	30	26	20	15
**Total teeth assessed**	2155	2092	2096	1996	1871	1616	1384	1079	912	675	505

### Periodontal health status at start of study

**Figure 1:**
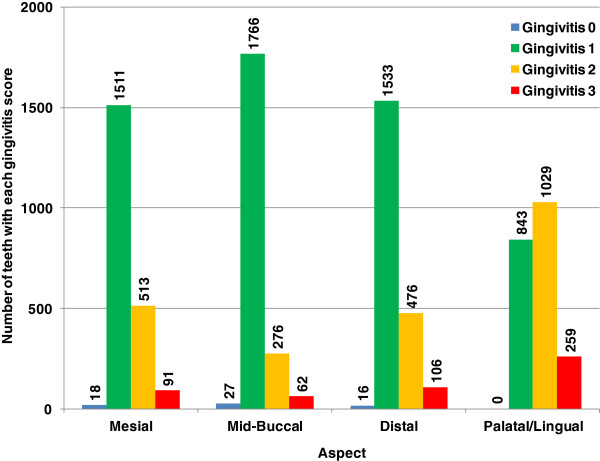
The number of teeth with each gingivitis score at first measurement, expressed by aspect.

One dog aged 2.5 years joined the trial with 6 teeth affected by periodontitis. Eleven dogs had either 1 or 2 teeth affected by periodontitis and were aged between 1.3 years and 6.9 years (mean 4.5 years). The 23 teeth identified as having periodontitis at the start of the study were not included in subsequent analysis.All of the dogs were observed to have gingivitis at the first assessment. Gingivitis was assessed on 4 individual aspects of each tooth. Of the 8526 measurements recorded 61 (0.7%) of the aspects were classified as healthy having no gingivitis or active periodontitis (gingivitis level 0 or G0) whilst 5653 (66.3%), 2294 (26.9%) and 518 (6.1%) aspects entered the study with gingivitis levels 1, 2 and 3 (G1, G2 and G3) respectively (Figure 1). No tooth was observed to be healthy around the whole gingival margin with 24.8% of the teeth having a maximum gingivitis level of 1, 57.4% having a maximum gingivitis level of 2 and 17.8% of the teeth having a maximum gingivitis level of 3.

### Incidence of periodontitis

**Figure 2:**
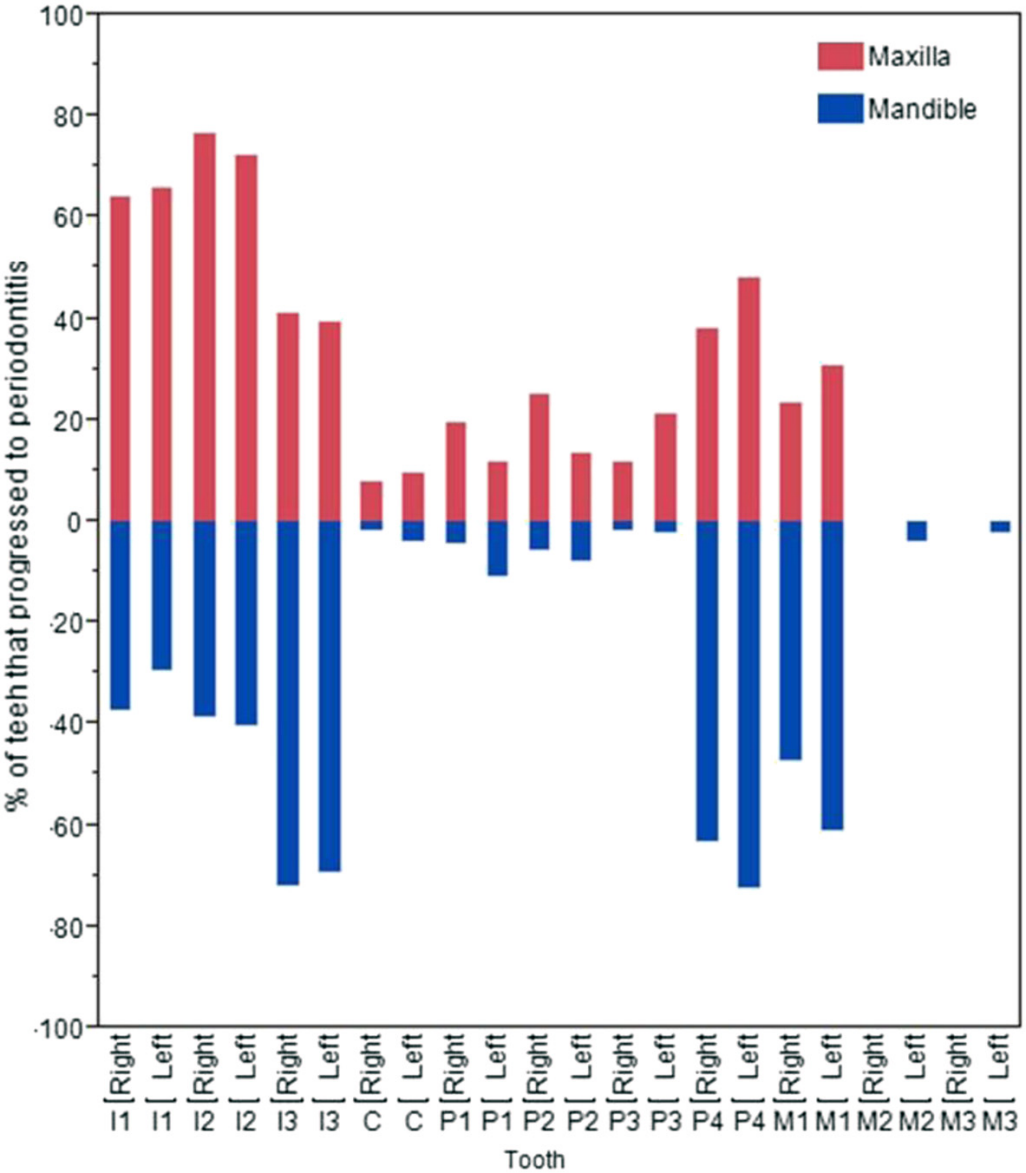
The percentage of each tooth which progressed to periodontitis.

Of the teeth that progressed to periodontitis (28.3% of the total teeth assessed), the incisors were the most represented tooth type and the canines the least represented (Table 2). When assessing individual teeth in the mandibles, the least affected teeth were the canines; the 1^st^, 2^nd^ and 3^rd^ premolars and the 2^nd^ and 3^rd^ molars which all had low levels of periodontitis (less than 11%). In contrast, all three incisors along with the 4^th^ premolar and the 1^st^ molar all showed high levels of periodontitis with between 29.4 and 72.5% of those tooth types affected. The same pattern was repeated on the maxillae, though the differences were less pronounced (Figure 2).

**Table 2:** Summary of teeth which progressed to periodontitis (not including periodontitis teeth at T0)

**Tooth type**	**Number of teeth at trial start**	**Sum of PD teeth**	**% of each tooth type which progressed to PD**	**% of total PD teeth**
**Incisor**	609	327	53.7	54.1
**Canine**	208	12	5.8	2.0
**Premolar**	808	180	22.3	29.8
**Molar**	507	85	16.8	14.1
**Total**	2132	604	28.3	100.0

To assess the position on the tooth where periodontitis occurred, each tooth was assessed around the whole gingival margin and data were recorded for four aspects of each tooth (Table 3). Only eight incidences of gingival recession or furcation exposure were observed throughout the study. Therefore, only the effect of increased probing depth was considered in this analysis.

**Table 3:** Percentage of each aspect that developed periodontitis out of all aspects for that tooth type

**Tooth type**	**Aspect**				
	**Mesial**	**Mid-Buccal**	**Distal**	**Combined buccal aspects**	**Palatal/Lingual**
**Incisor**	4.27	0	7.22	11.49	44.99
**Canine**	2.4	1.44	1.92	5.76	0.96
**Premolar**	3.47	1.36	13.12	17.93	8.17
**Molar**	10.85	1.24	0.99	13.08	8.44

**Figure 3:**
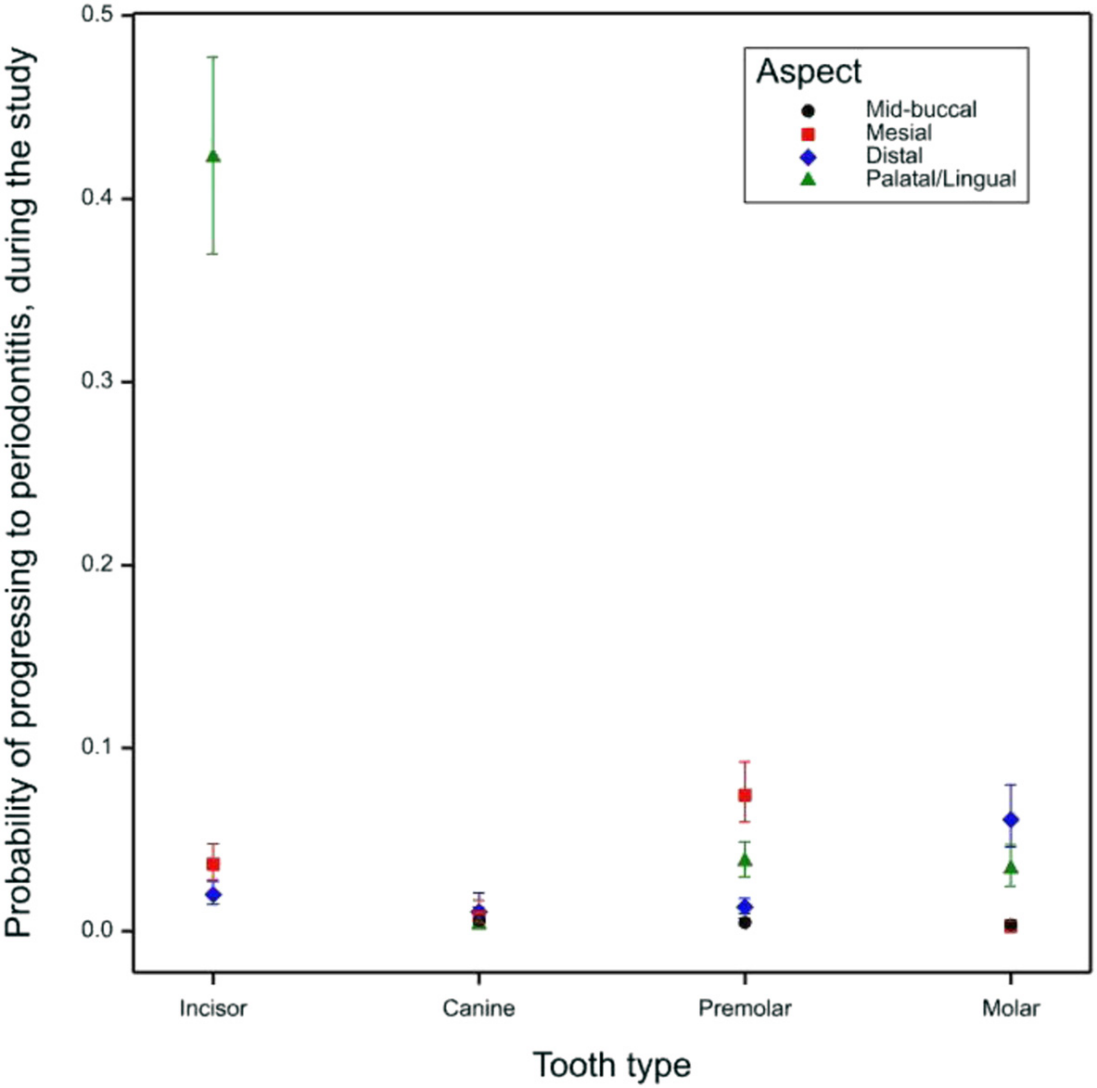
**Probability of each aspect progressing to periodontitis.** Error bars signify 95% confidence intervals.

The palatal/lingual aspect of the incisors had a significantly higher proportion of periodontitis compared to other aspects (*p* < 0.001). The premolar teeth were significantly more likely to be affected on the distal aspect (*p* < 0.001) whilst the molar teeth had a significantly higher proportion of periodontitis on the mesial and palatal/lingual aspects compared to the mid-buccal and distal aspects (*p* < 0.001) (Figure 3). The majority of this difference was driven by the high level of increased probing depths observed at the point where the distal aspect of 4^th^ premolar meets the mesial aspect of the 1^st^ molar in both maxillary and mandibular quadrants.

### Rate of periodontitis disease progression

**Figure 4:**
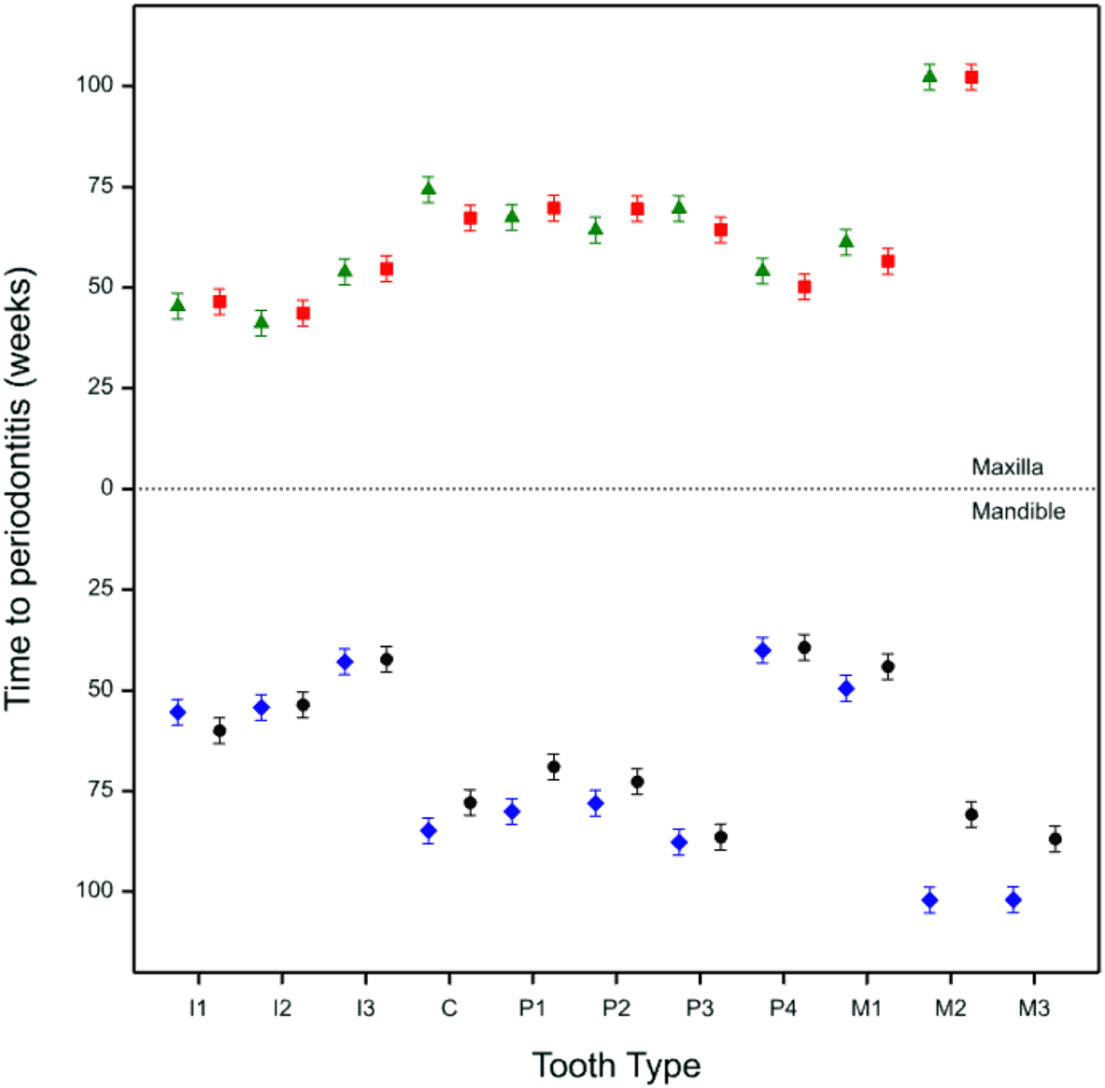
**Estimated time for each tooth to progress to early stage periodontitis.** Green triangles and blue diamonds = right-hand side of the mouth; red squares and black circles = left hand side of the mouth. Error bars signify 95% confidence intervals.

Rates of periodontitis disease progression were modelled for each tooth type (Figure 4). There were a number of teeth that were estimated to have statistically significant differences in their rates of progression. For example, the predicted time it takes the mandibular 4^th^ premolars to progress to periodontitis was significantly lower than any of the other mandibular premolars (*p* < 0.001) and the 1^st^ mandibular and maxillary molar teeth were significantly more likely to progress than all of the other molar teeth (*p* ≤ 0.007). The maxillary 2^nd^ molars and the right mandibular 2^nd^ and 3^rd^ molars had no observed periodontitis and the model predicted that they would require 102 weeks to reach the disease state.

### Relationship between age and the rate of periodontitis progression

**Figure 5:**
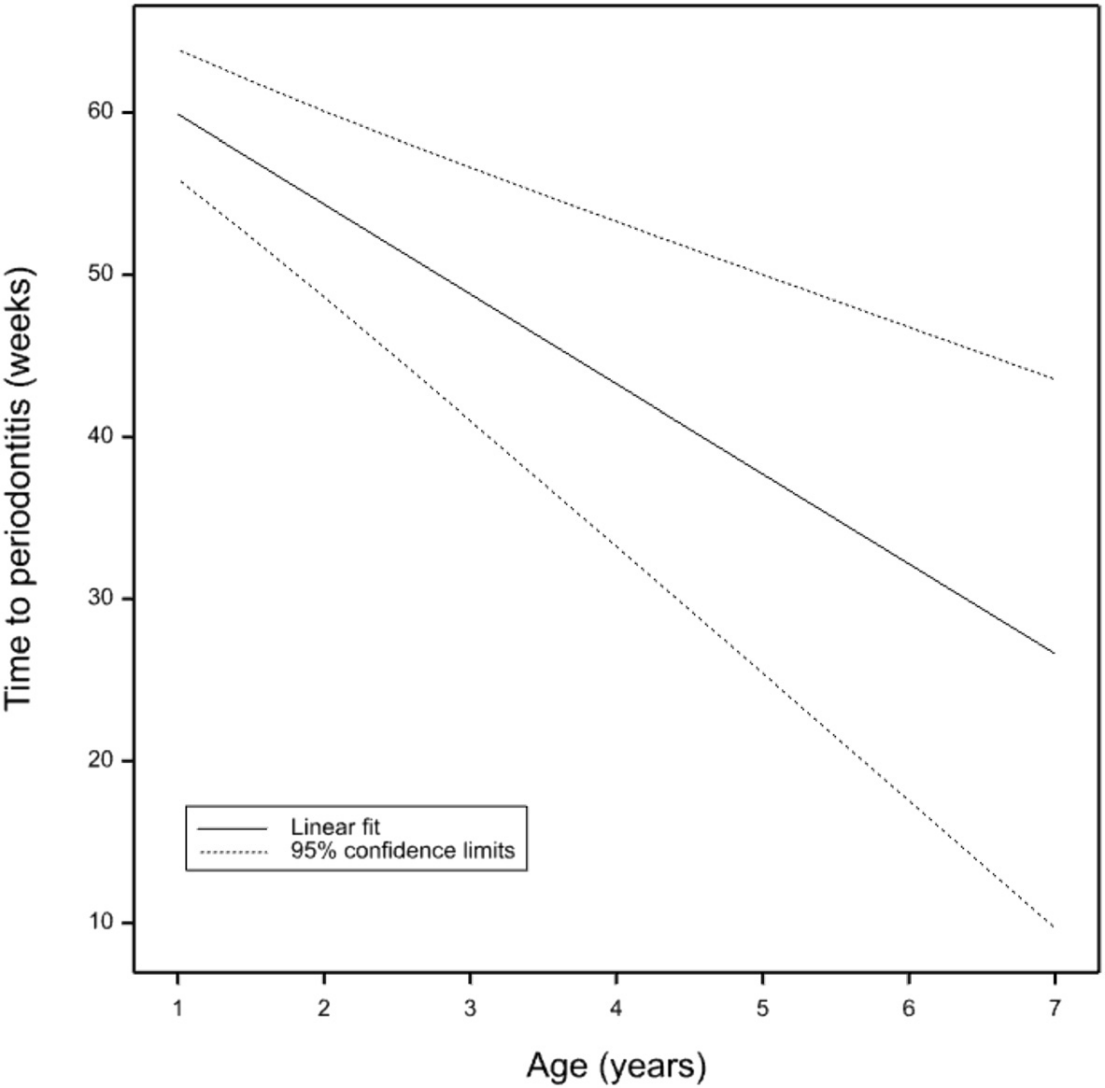
Estimated linear fit of age against time to periodontitis with 95% confidence limits.

There was a significant linear effect of age on the time it takes the teeth to progress to periodontitis. With every year’s increase in age there was a reduction in time to periodontitis of 5.5 (s.e. 1.21) weeks (Figure 5) demonstrating that as dogs age they progress to periodontitis significantly faster than younger dogs.

### Relationship between gingivitis and periodontitis

**Figure 6:**
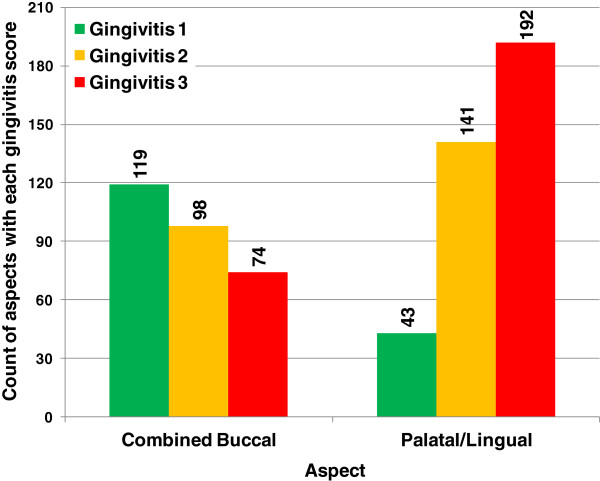
Histogram of gingivitis scores for each aspect of the tooth affected with periodontitis.

The distribution of gingivitis scores on the aspects of the teeth affected by periodontitis are shown in Figure 6 and Table 4. It is clear that the palatal/lingual and buccal aspects behave quite differently. On the palatal/lingual aspect, periodontitis is often associated with later stage gingivitis (mostly levels 2 and 3) whereas no such relationship exists for the combined buccal aspect where the highest proportion of teeth that progressed to the early stages of periodontitis were observed to have gingivitis level 1.

**Table 4:** Percentage of aspects with each gingivitis score, for aspects affected with periodontitis

**Aspect**	**Gingivitis score**			
	**1**	**2**	**3**	**Total**
**Mesial**	7.65	6.15	3.30	17.09
**Mid-Buccal**	1.35	1.05	0.45	2.85
**Distal**	8.85	7.50	7.35	23.69
**Combined Buccal aspects**	17.84	14.69	11.09	43.63
**Palatal/Lingual**	6.45	21.14	28.79	56.37

**Figure 7:**
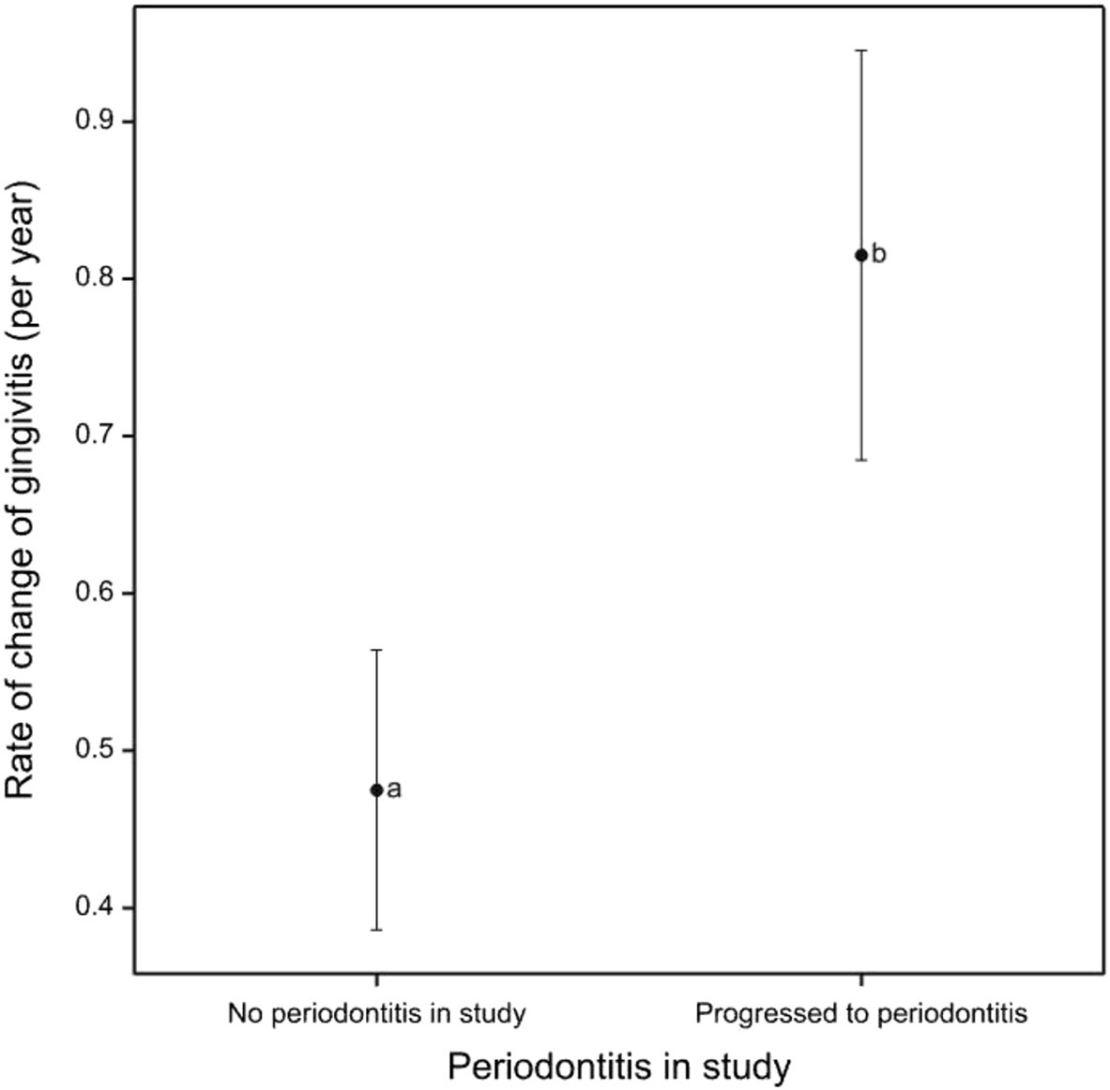
**Gingivitis score progression rate according to periodontitis progression.** Mean rate of gingivitis score progression per year in periodontitis affected aspects compared to aspects with no periodontitis progression, with 95% confidence intervals.

To further explore the relationship between gingivitis and periodontitis the progression rate of gingivitis was assessed. There was a significantly faster rate of gingivitis progression for teeth that progressed to periodontitis compared to those teeth that did not (*p* < 0.001) (Figure 7).

**Figure 8:**
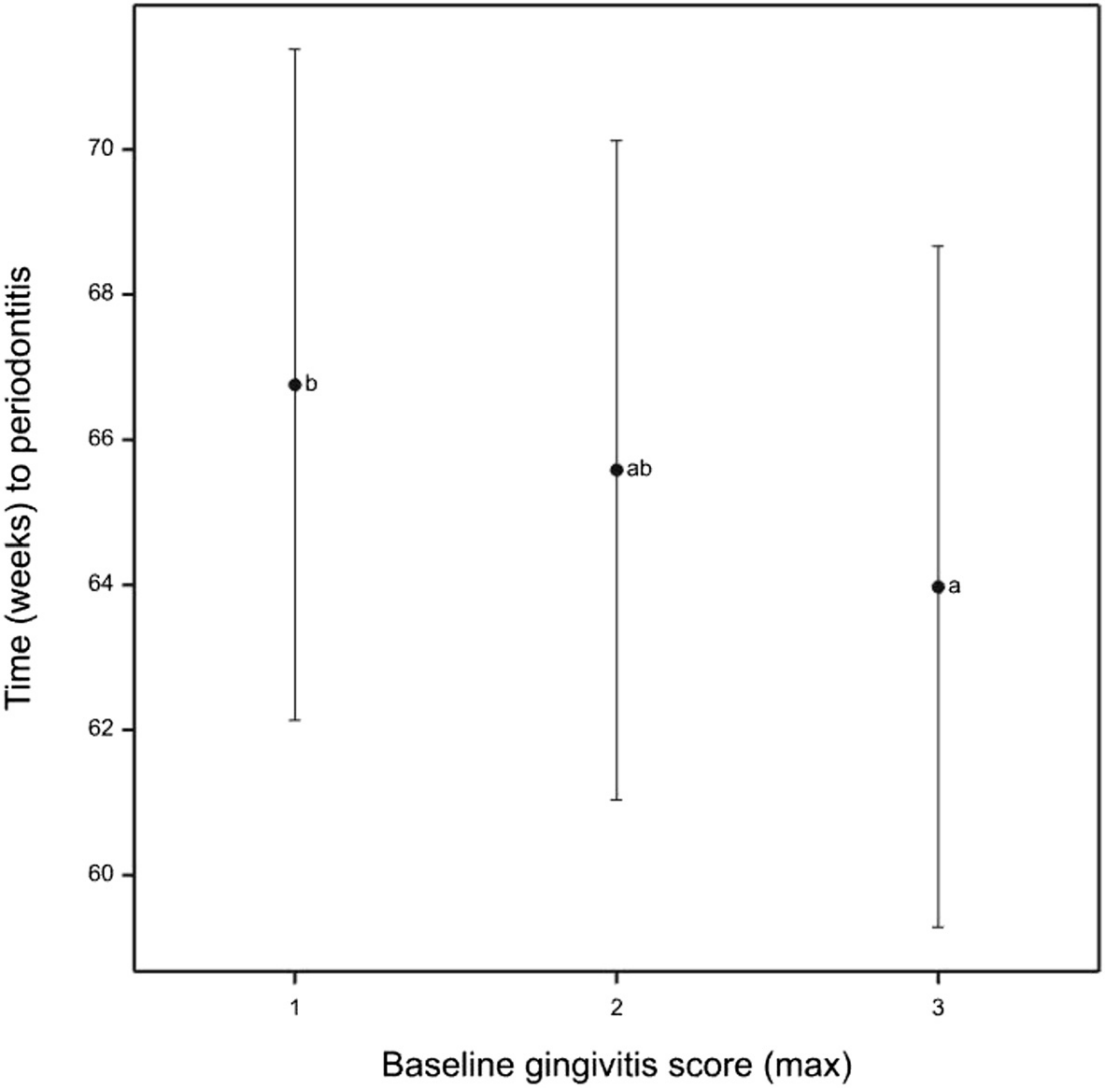
**Average time to periodontitis by starting gingivitis score.** Estimated average time to periodontitis for teeth by baseline maximum gingivitis scores with 95% confidence intervals. Letters represent Tukey HSD homogenous groups at the 5% level.

To determine whether the gingivitis score was a predictor of future time to periodontitis the starting scores for each tooth were examined to see whether they influenced the likelihood of progressing to periodontitis in the trial. Table 5 shows that similar proportions of teeth developed periodontitis regardless of the starting gingivitis score. This was supported by modelling the time it takes for each tooth to develop periodontitis for each starting gingivitis state. Although this showed that the time that teeth took to progress to periodontitis starting from a baseline gingivitis score of 1 was significantly different to teeth starting at gingivitis level 3 (*p* = 0.011) (Figure 8), the difference in rates was actually very small. Teeth starting with gingivitis level 1 would take on average 66.8 weeks to develop periodontitis whilst teeth starting at gingivitis levels 2 and 3 would take 65.6 and 64.0 weeks, respectively.

**Table 5:** Numbers and proportions of teeth progressing to periodontitis compared to starting gingivitis score of teeth

**Starting gingivitis score**	**Number at start**	**Number of teeth which developed periodontitis**	**Percentage of teeth which developed periodontitis**
**G1**	528	114	21.59
**G2**	1224	370	30.23
**G3**	380	120	31.58

## Discussion

This study describes the incidence and progression of periodontitis in 52 miniature schnauzers based on full mouth examinations over a period of up to 60 weeks. Dogs as young as 1.3 years were affected by the early stages of periodontitis. Only one dog did not develop periodontitis in any teeth during the course of this study and 35 dogs developed the early stages of periodontitis in 12 or more teeth within 60 weeks of stopping the oral hygiene programme. Numerous studies have demonstrated a correlation between age, prevalence and severity of periodontal disease [2-6]. This study has also shown that as dogs age they progress towards periodontitis more quickly than younger dogs when efforts at maintaining oral hygiene are stopped.

Previous studies have reported prevalence rates of periodontitis ranging from 44 to 63.6%, in mixed age populations [1-4], rising to 84% in dogs aged 3 years or older [2], 82.3% when dogs reach the age of 6 years or older [4] and in one study, periodontitis was present in 100% of poodles over the age of 4 years [5]. In this study 98% of dogs had developed some level of periodontitis within 30 weeks of stopping toothbrushing. This high record of incidence is likely to be a consequence of 3 factors. Firstly, every single tooth in every dog was analysed around the whole of the gingival margin; secondly the breed studied (miniature schnauzer) is known to be a high risk breed for periodontitis (internal data – unpublished) and thirdly the very earliest stages of periodontitis were measured.

In this study, preventative tooth brushing was stopped either one week or 18 weeks prior to the first measurement. The dogs that had tooth brushing stopped 18 weeks earlier were used in a pilot study to assess the optimal time interval for dental assessments to correctly determine periodontitis progression. All dogs started the trial with some level of gingivitis (0.7% of aspects were healthy). This therefore suggests that gingivitis develops rapidly even in young dogs of this breed (minimum age 1.3) when an oral care regime is removed. This is consistent with a previous longitudinal study in beagle dogs, where all dogs exhibited gingivitis within 2 weeks of tooth brushing being removed (control dogs who received twice-daily tooth brushing showed no gingivitis) [9]. Consistent with other studies, gingivitis did not always lead to periodontitis in this study [12-14].

Rates of progression were estimated for each tooth and tooth type by modelling the time that each takes to progress to periodontitis (Figure 4). Unsurprisingly, the teeth that had the highest incidence levels also had the fastest estimated rates of progression. This model suggests that the incisors, 4^th^ premolars and 1^st^ molars are the teeth that develop periodontitis the fastest. The 110, 210, 410 and 411 molar teeth, for example, did not develop periodontitis in this study and were modelled as requiring 102 weeks to progress. A further study of a design that allows all teeth to progress to periodontitis would be required to validate these model estimates.

There was a low incidence of periodontitis in the canines and rostral premolars and it was rarely observed in the 2^nd^ and 3^rd^ molars. These results are consistent with a study of a population of 162 dogs of various breeds in which the molars and distal premolars were more often affected by periodontal lesions compared to the rostral premolars and canines [4]. In contrast, in a population of 123 poodles the highest incidence of periodontitis was in the canine teeth [5]. However, in the poodle study, the highest rate of missing teeth was in the incisors and first premolar teeth P1 which may indicate high levels of periodontitis-related tooth loss. In this work dogs were removed from the study if they developed the early stages of periodontitis in 12 or more teeth and therefore incidence rates are likely to be underestimated since not all teeth had the same opportunity to progress to periodontitis. The decision to remove dogs from trial that had 12 or more periodontitis affected teeth was made to balance the need to have sufficient numbers of most tooth types progressing to periodontitis without compromising the welfare of the dog. To determine the actual incidence rate for tooth types that progress more slowly, a study design is required in which every tooth type is able to progress to periodontitis. This study design would be likely to result in the teeth that have more rapid progression rates reaching the later stages of periodontitis (PD2 and above). This design does not fit within our ethical or animal welfare policies and so was not pursued.

In a previous study by Lindhe *et al.*[9] the upper premolars and molars exhibited increased loss of attachment compared to the lower premolars and molars in Beagle dogs [9]. In contrast, in this study the lower distal premolar and rostral molar teeth exhibited increased incidences of periodontitis compared to the same teeth on the maxilla. The aspect which was most frequently associated with periodontitis on the 4^th^ premolar teeth in this study was the distal aspect of the buccal surface, whilst the 1^st^ molar teeth were most affected on the mesial aspect. This may be due to a build up of plaque between these two teeth which may in turn lead to pathology. There is some evidence that calculus build up is greater on the maxillary 4^th^ premolar and 1^st^ molar in multiple breeds of dogs [6] and the position of the parotid and zygomatic salivary duct openings has been suggested as a probable factor for the increased levels on the upper 4^th^ premolar [15]. Calculus wasn’t measured in this study as it isn’t considered the primary aetiological factor in periodontitis [16] so a direct comparison of calculus build up and disease progression cannot be made. The reason for this difference between tooth types is unclear but may be related to the way that dogs in this breed eat their food or maybe due to salivary flow behind the incisors. These potential relationships are yet to be investigated.

The majority of the periodontal pockets observed in this study occurred on the palatal/lingual aspect of the teeth (Table 3). This initially appears to contrast with a previous study of 408 dogs of various breeds, size and age, which observed that the buccal surface of the teeth was more affected than the palatal/lingual surface [3]. However a closer inspection of the data reveals that the vast majority of the periodontitis found on the palatal/lingual aspect occurs on the incisors and that on the other tooth types (canines, molars and premolars) periodontitis was more commonly associated with the buccal aspect (Table 3).

Taken together, these differences between the teeth and aspects affected in different studies suggest that there are breed differences that determine which teeth are most likely to develop periodontitis. It has previously been documented that brachycephalic breeds and dogs with tooth overcrowding are more vulnerable to periodontitis [7]. Tooth overcrowding in the incisor region of miniature schnauzers may explain why a high incidence of periodontitis was observed in this study. The close proximity of these teeth to each other may allow transfer of disease-associated plaque bacteria and thus increase the burden of disease. Similarly, the overlap between the distal premolar P4 and the rostral molar M1, may lead to a build of plaque and ultimately disease. In addition to plaque potentially causing disease in this region, the close proximity of these two teeth increases the likelihood that any loss of attachment or bone may affect both teeth.

The incidence of periodontitis and estimated progression rates differed for every dog, tooth type and aspect, consistent with previous studies which suggest that periodontal lesions do not progress at the same rate [9,17]. For example, one dog aged 3.5 years did not develop any periodontitis during the study. Another dog aged 6.8 years only developed periodontitis in 2 teeth in the 60 week time frame despite four of his siblings developing periodontitis in 12 or more teeth within 42 weeks.

There was a significant linear effect of age on the time it takes the teeth to progress to periodontitis in this study. With every year’s increase in age there is a reduction in time to periodontitis of 5.5 (s.e. 1.21) weeks (Figure 5). It has previously been reported that a positive correlation exists between increasing age, and the prevalence and severity of periodontal disease [6,7,11,14]. This study has shown that older dogs progress to periodontitis faster than younger dogs. The reason/s for these differences are not clear. One could hypothesise that the response of the immune system to the bacteria alters with age. Lowered immunity in aged subjects, or immunosenescence, is an accepted phenomenon in humans as is increased proinflammatory status which is believed to be a causal factor in increased mortality rates in the elderly [18]. Changes in immune parameters with age in dogs have been reported in Labrador retrievers ranging in age from 0.8 to 11.5 years and 2 to 10 years [19,20] and fox terriers mean age 1.8 years compared to mean age 11.5 years [21]. Breed differences have also been proposed between German shepherd dogs and Labradors [22]. It has been reported that these changes in the immune response are similar to that seen in humans and contribute to the ageing process in dogs [23]. Therefore, older dogs may have increased severity in periodontitis because they have either been affected for longer or because their immune system can either no longer cope with the pathogenic attack or develops an excessive inflammatory response.

A number of studies have challenged the previously held view that gingivitis always progresses to periodontitis [24]. Here we have reported that teeth that progress to early stage periodontitis have a more rapidly progressing gingivitis score compared to teeth that do not progress to periodontitis. This could suggest a relationship between gingivitis and periodontitis. However, in teeth that progress to periodontitis the rate of progression of gingivitis is slow. When the length of this trial is considered (60 weeks) it becomes clear that the speed of gingivitis progression that was observed was not rapid compared to progression towards periodontitis. Furthermore, only 31.58% of teeth that were scored as G3 at baseline progressed to periodontitis which was similar to teeth that were scored as G1 at baseline (21.59%). In addition, the predicted rate at which teeth would develop periodontitis was similar regardless of whether the teeth started in gingivitis level 1 or 3. Finally we have reported that on buccal aspects, when periodontitis was first detected the associated gingivitis score was more likely to be G1 than G3. However in contrast, on palatal aspects there was a greater tendency for periodontitis to be associated with higher gingivitis scores.

Taken together the data reported in this study indicate that there is only a weak relationship between gingivitis and progression to periodontitis. Of particular significance is the fact that the current gingivitis state of a tooth does not predict the time that tooth will take to get periodontitis. This is consistent with human studies which reported that most sites of gingivitis did not progress to periodontitis [24]. The lack of a strong relationship between gingivitis and periodontitis may result from the inherent variability seen in the method. The gingivitis score relies heavily on one particular feature, time to bleeding. It is not known if there is any biological significant difference between bleeding that happens slightly delayed or immediately and yet one gives a score of 2 and the other 3. The technique is also subjective in nature which adds to the noise. To effectively assess gingival and periodontal health a more objective method is therefore required that relies on biological markers of disease progression that are more sophisticated than simple time to bleeding or gross inflammation. An improved understanding of the biological changes in the canine immune response and the bacterial population in the oral cavity that occurs during progression towards periodontitis would undoubtedly be a good first step.

This longitudinal assessment has reported the estimated rates of progression of periodontitis in different tooth types in adult miniature schnauzers. The effects of age, tooth type and aspect observed in this study were identified over and above the noise in the data due to veterinary treatment and diet. The teeth most likely to progress to periodontitis in this breed are the incisors, 4^th^ premolars and 1^st^ molars. It is clear that not every dog or indeed every tooth within a dog is equally affected. This study has highlighted the value of whole mouth, site specific examinations over time. Over half of the periodontal pockets detected were on the lingual or palatal aspect of the tooth. This supports the evidence that examining a dog with a conscious assessment of gum health on the buccal surface is insufficient to diagnose periodontal disease.

Miniature schnauzers in this study developed periodontitis from a young age (as young as 1.3 years) and the likelihood of developing the disease increased with age. The disease typically progressed on aspects of the teeth that cannot be readily seen in a conscious oral examination. Based on the results of this study, in the absence of an effective oral care regime periodontitis will develop rapidly in this breed and if left unchecked could progress further and ultimately lead to loss of teeth and significant discomfort for the dog [7,16]. Periodontitis has also been associated with a number of serious systemic diseases such as endocarditis and renal disease in humans [25] and renal, hepatic and cardiac disorders in dogs [13]. It is therefore of great benefit to maintain dogs in good periodontal health which for this breed requires a regular oral care regime and ideally frequent dental assessments. Given the rate of development of periodontitis observed in this study, dental assessments need to be at least twice yearly to have the best chance of catching the disease in its earliest stages. It is reasonable to assume that twice yearly dental assessments of other small and toy breeds with similar periodontitis incidence rates are likely to be beneficial.

## Methods

### Dogs

Fifty two miniature schnauzers aged between 1.3 and 6.9 years, with an average bodyweight of 8.8 kg (range 6.6 – 11.4) and housed at the WALTHAM® Centre for Pet Nutrition were enrolled in this study. Twenty eight dogs were female (at the start of the trial entire n = 9, neutered n = 19) and 24 were male (all neutered). Two of the bitches were subsequently spayed some 18 and 42 weeks into the study. All dogs had been on an oral care regime of toothbrushing every second day since approximately 1 year of age. Toothbrushing was stopped between one week (n = 42 dogs) and 18 weeks (n = 10 dogs) before the first dental assessment. The latter group had been on pre-trial study to determine the optimal time window for making dental assessments. The date of the final intervention was used as time zero in the statistical models assessing disease progression rates. All dogs received a pre-study veterinary examination to ensure suitability for trial, which included a physical examination, routine blood work and an assessment of the dog’s veterinary history.

This study was approved by the WALTHAM® Ethical Review Committee and run under licensed authority in accordance with the UK Animals (Scientific Procedures) Act 1986.

### Dental assessment procedures

Dental assessments were performed under general anaesthesia. Following a pre-medication of acepromazine (0.05 mg/kg) and buprenorphine (0.02 mg/kg), general anaesthesia was induced by an injection of propofol (4 mg/kg) via an intravenous catheter. Gaseous anaesthesia was maintained with oxygen and isoflourane via a cuffed endotracheal tube.

Six people were used for the dental scoring assessments. The scorers were all trained by a Recognised European Specialist in Veterinary Dentistry (LM) and then calibrated 2 weeks prior to the start of the trial to ensure consistency between scorers. During the course of the trial all scorers were re-assessed at least every 3 months to ensure consistency was maintained across time. Each dog was assessed by the same scorer throughout the whole trial (with a minor number of unavoidable exceptions for illness etc.). Scorers were not permitted to review previous results and in effect were assessing the dogs blind. In addition, a second scorer was also present to confirm or reject the depth of a periodontal pocket if required.

All teeth were scored individually at each assessment. Each measurement was taken at the gingival margin using a periodontal probe. A gingivitis score between 0 and 4 was recorded for the mesial, mid-buccal, distal and palatal/lingual aspect of each tooth using a modified combination of the gingival index (GI) and sulcus bleeding index (SBI) [26]. Probing depth, gingival recession and furcation exposure were recorded according to the criteria in Table 6. Probing depth was measured from the gingival margin to the bottom of the periodontal pocket. Gingival recession was measured from the cementoenamel junction (CEJ) to the gingival margin using the graduations of a periodontal probe. Total attachment loss was calculated as the sum of the gingival recession and the periodontal probing depth in accordance with established protocols [12,16]. No evidence of gingival hyperplasia was observed. In this study, where the very early stages of periodontitis were identified, total attachment loss was the result of an increase in periodontal probing depth except on 8 occasions; 4 of which were incidences of gingival recession and 4 of which were when furcation exposure was observed. Periodontitis stage 1 (PD1) was classified as being up to 25% attachment loss and periodontitis stage 2 (PD2) as between 25 and 50% attachment loss. Each dog was assessed every six weeks +/− 1 week for up to 60 weeks.

**Table 6:** **Miniature schnauzer periodontal disease scoring system (adapted from Wiggs & Lobprise,**[26]**)**

**Score**	**Gingivitis**	**Periodontal probing depth (mm)**	**Gingival recession (mm)**	**Furcation exposure**
**Health (G0)**	No gingivitis, pink (or pigmented) healthy gingiva, no inflammation and no bleeding on probing	<1	0	0
**G1**	Very mild gingivitis (red, swollen but no bleeding on probing)	≥1	0	0
**G2**	Mild gingivitis (red, swollen and delayed bleeding on probing)	≥1	0	0
**G3**	Moderate gingivitis (red, swollen and immediate bleeding on probing)	≥1	0	0
**G4**	Severe gingivitis (ulceration, spontaneous haemorrhage, profuse bleeding on probing)	≥1	0	0
**PD1**	G1-G4: gingivitis must be present (i.e. active periodontitis)	>2 (>3 on canine teeth)	>0	Grade 1; feel an indentation between the roots and the probe may advance 1 mm.
**PD2**	G1-G4: gingivitis must be present (i.e. active periodontitis)	>4 (>6 on canine teeth)	>2 (>3 on canine teeth)	Grade 2; obvious indentation between the roots and probe advances 50%.

**Figure 9:**
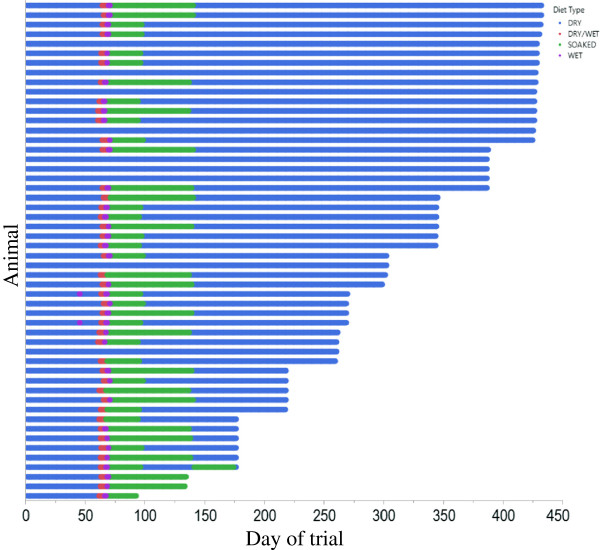
Bar chart showing the diet types fed to each animal for the duration of the trial.

To avoid teeth progressing to the later stages of PD, they were scaled and polished as soon as periodontitis was detected and then no longer included in the study. These periodontitis teeth were scaled and polished at every subsequent assessment until the dog left the trial. The variable rate of periodontitis progression resulted in 12 teeth reaching PD2 at the first time they were detected as having periodontitis. These teeth were scaled and polished in the same way as teeth that had reached PD1 and were also included in the analysis of the results. No teeth progressed past PD2 during the course of the trial. If a dog developed periodontitis in 12 or more teeth it received a full mouth scale and polish, was removed from the study, and the oral care regimen of toothbrushing was reinstated. These oral care criteria were selected to prevent any teeth progressing to later stages of periodontal disease. An earlier study (data not published) had shown that placing dogs at an early stage of periodontitis on to a toothbrushing regime following a full mouth scale and polish would maintain their oral health status and prevent further disease progression.Dogs were routinely maintained on a dry kibble diet. Some of the dogs were on nutritional research studies and on occasion they were fed diets that were either a mixture of dry and wet, dry soaked in water or a solus wet diet (Figure 9). Across all of the dogs 83% of the feeding events were of a solus dry diet with a range of 46% to 100% in individuals. Routine veterinary care was permitted throughout the study as required, which on occasions included administration of antibiotics and anti-inflammatory drugs. Records of these and other veterinary treatments were maintained for each dog.

### Statistical methods

The probability of progressing to periodontitis was analysed by generalised linear mixed models, for binary data, with tooth nested in dog as the random effects and aspect, tooth type and their interaction as the fixed effects. A significance level of 5%, after Bonferroni correction, was used to compare mean probabilities (within tooth type, between aspects and within aspects between tooth types). Due to the censored nature of the data the estimated probabilities are likely to be underestimates of the real life progression rates. Prior to this analysis the teeth that had periodontitis at baseline measurement were removed.

The time to progress to periodontitis was estimated by censored ANOVA analyses [27]. This analysis allows incorporation of the teeth that were taken off study before they were allowed to progress (i.e. censored observations). The censored observations also included those teeth which did not progress to periodontitis because the dog was removed from trial before the entire dog’s dentition developed periodontitis. The final model incorporated tooth nested in dog as the random effects, tooth and baseline gingivitis score as categorical fixed effects and age as covariate. Comparisons between estimated mean times to progress to periodontitis were made using Tukey HSD tests at the 5% level. The model building also investigated the fixed effects of gender, diet format, percentage of measurements on a veterinary treatment and plausible two-way interactions, however these were dropped from the model due to lack of significance, with *p* > 0.05.

The rate of change of gingivitis was estimated for each aspect (that had at least 4 time points) of each tooth by linear regression with time. The resulting rates of change were analysed by linear mixed models, incorporating aspect nested in tooth nested in dog as random effects and tooth number, periodontitis status at the end of the study, aspect as fixed effects, along with tooth type, jaw and their interaction. The model building also investigated fixed effects of age, gender, diet format, percentage of measurements on a veterinary treatment and plausible two-way interactions, however these were dropped from the model due to lack of significance, with *p* > 0.05.

Analyses were performed using GenStat v14 statistical software [28].

## Abbreviations

CEJ: Cementoenamel junction; G0: Gingivitis level 0 (healthy gingiva); G1: Gingivitis level 1 (very mild gingivitis); G2: Gingivitis level 2 (mild gingivitis); G3: Gingivitis level 3 (moderate gingivitis); G4: Gingivitis level 4 (severe gingivitis); GI: Gingival index; PD: Periodontal disease; PD1: Periodontitis stage 1 (up to 25% attachment loss); PD2: Periodontitis stage 2 (25-50% attachment loss); SBI: Sulcus bleeding index.

## Competing interests

Mark Marshall, Corrin Wallis, Alison Colyer and Stephen Harris work for Mars Petcare.

## Authors’ contributions

MDM – participated in the design and co-ordination of the study, maintained data integrity, drafted and prepared the manuscript; CVW – participated in the design and co-ordination of the study, drafted and reviewed the manuscript; LM – participated in the design of the study, assisted with clinical scoring methodologies, assisted with results interpretation and reviewed the manuscript; AC – participated in the design of the study, performed all statistical analyses and reviewed the manuscript; ADT – built the database for capturing all data, participated in study modification, maintained data integrity and delivered the final dataset; SH – conceived and participated in the design and co-ordination of the study, reviewed the results and reviewed the manuscript. All authors have approved the final article.
